# Apolipoprotein E Genetic Polymorphism and Stroke Subtypes in a Bangladeshi Hospital-Based Study

**DOI:** 10.2188/jea.11.131

**Published:** 2007-11-30

**Authors:** Anisul Haque Chowdhury, Tetsuji Yokoyama, Yoshihiro Kokubo, Mohammad Mostafa Zaman, Anisul Haque, Heizo Tanaka

**Affiliations:** 1 Medical Research Institute, Tokyo Medical and Dental University, Tokyo, Japan.; 2 Rheumatic Fever and Heart Diseases Control Center, Dhaka, Bangladesh.; 3 Department of Neurology, Bangabandhu Sheikh Mujib Medical University, Dhaka, Bangladesh.

**Keywords:** ApoE genetics age-dependence stroke Bangladesh

## Abstract

The association between apolipoprotein E (apoE) genetic polymorphism and stroke has not been concordant in different racial populations. We investigated the association between apoE genotypes and stroke subtypes by a case-control study in Bangladesh for the first time among south Asian countries.

First-ever-stroke patients (n=227; cerebral infarction, n=147, cerebral hemorrhage, n=80) and 190 controls were recruited from a hospital in Dhaka, Bangladesh. The diagnosis of stroke was based on CT and clinical findings. Cerebral infarction was classified anatomically into cortical and penetrating region. Infarction in the cortical region was further categorized etiologically into thrombosis and embolism. Cerebral hemorrhage was considered as a whole in all analyses. ApoE genotypes were determined by restriction fragment length polymorphism.

In the multivariate conditional logistic regression analysis adjusted for potential confounders both the *ε*3/*ε*4 genotype and *ε*4 carrier conferred an approximately 3-fold increased risk for cerebral thrombosis in the cortical artery region (OR 3.5, 95% CI 1.2 to 10.4 and OR 3.1, 95% CI 1.1 to 9.0, respectively) compared with *ε*3/*ε*3 genotype. However, when the analysis was restricted to the elderly (>60 years), *ε*2 carrier was associated with a risk of hemorrhagic stroke (OR 19.2, 95% CI 1.3 to 295.2).

Our study suggested that both apoE *ε*3/*ε*4 genotype and *ε*4 carriers were risk factors for cerebral thrombosis in cortical artery region, whereas *ε*2 carrier was a risk factor for hemorrhagic stroke in the elderly.

## INTRODUCTION

Stroke is a leading cause of severe disability and mortality in both developed and developing countries^1^^)^. It is a complex condition influenced not only by environmental risk factors but also by genetic factors^2^^, ^^3^^)^. Among the potential genetic factors, in this study, we have focused on the apoE genetic polymorphism which plays a crucial role in triglyceride rich lipoprotein catabolism and cholesterol homeostasis^3^^, ^^4^^)^, and thus in the risk of stroke. Among three codominant alleles of apoE gene (*ε*2, *ε*3 and *ε*4) the protein products of *ε*2 and *ε*4 alleles have separate and opposite influences on plasma lipoprotein concentration with higher total (TC) and low density lipoprotein (LDL-C) cholesterol concentration in *ε*4, (intermediate in *ε*3) and lower in *ε*2 carriers^5^^-^^7^^)^.

Several studies documented a positive association between apoE *ε*4allele and early development of atherosclerosis^8^^, ^^9^^)^, and ischemic heart disease^10^^, ^^11^^)^, and the association was confirmed in both sexes by a meta-analysis^12^^)^. In other studies, however, the role of apoE in stroke occurrence yielded conflicting results. Pedro-Botet et al^13^^)^ first reported that the variant *ε*4 of apoE was positively associated with ischemic stroke in men. Afterwards, other case-control studies either disputed^14^^-^^18^^)^ or supported^19^^-^^21^^)^ this finding. For example, Kessler et al^19^^)^ found *ε*2 allele to be more common in the large-vessel atherosclerosis subtype of ischemic stroke in a German population. Of three population based studies one of which^22^^)^ detected the protective effect of *ε*2 in an older population, while the remaining studies^23^^, ^^24^^)^ did not identify apo E as a risk factor for over-all stroke. These inconsistencies may be due to population heterogeneity, diversity in stroke classification, different age range, as well as small sample sizes. Of these, diversity in stroke classification renders major difficulty to interpret the results of the studies because stroke is a pathogenically and phenotypically heterogeneous disease with the role of risk factors for a stroke subtype differing from other subtypes. Most of the previous studies failed to subdivide stroke patients according to their pathogenesis.

Thus, we conducted a case-control study of stroke with a distinct CT classification in a Bangladeshi hospital so that we could test the hypothesis that the apoE genetic polymorphism was related to stroke differently according to its subtypes.

## MATERIALS AND METHODS

### Study subjects

The present design was a hospital-based case-control study of stroke. A total of 291 consecutive stroke patients were admitted to the neurology and medicine units, Dhaka Medical College Hospital (DMCH), Dhaka, Bangladesh, during the period from April 1998 through February 1999. DMCH is one of the largest public general hospitals in the country providing easy access to people from all socio-economic classes. Among the stroke patients, CT findings were obtained for 262 patients. Of the CT documented patients, 4 clinically potential cerebral infarction cases failed to demonstrate any lesion on CT scan, 19 suffered recurrent stroke and 12 subjects refused to participate. 227 first-ever-stroke cases were finally recruited in the present study according to the following criteria.

*Cases:* Stroke patients fulfilling standard World Health Organization criteria^25^^)^ for diagnosis with documented CT findings were considered as cases.

The patients were categorized into stroke subtypes on the basis of CT findings, clinical histories and examinations. Cerebral infarction (If low density area on CT image) was categorized anatomically into cortical and penetrating infarctions. Cortical infarction was further classified etiologically into (1) cortical thrombosis, if the onset of stroke had been gradual and in the absence of any source of embolism.; (2) embolism, If either CT image had shown hemorrhagic infarct or sudden onset of focal neurological symptoms had been accompanied by any one of the following sources of embolism: (a) atrial fibrillation on electrocardiogram; (b) presence of heart murmur or carotid bruit assessed clinically, and (c) history of recent myocardial infarction. We failed to assign 6 cortical infarctions into either group and thereby considered them as of undetermined type.

Cerebral hemorrhage, which excluded subarachnoid hemorrhage, was confirmed by the presence of high-density area on CT image.

CT scan was performed between 3 and 7 days after stroke onset for all cases. We could perform only one CT scanning for each case with cerebral infarction.

*Controls*: Since it was very difficult to get healthy controls from free-living populations in Bangladesh, idiopathic cataract patients in good general conditions who were admitted to the eye department at DMCH for elective surgery during the same period were recruited as controls. The controls did not give a prior history of stroke and were free from clinically detectable cerebrovascular disease. Of the 201 cataract patients, 190 consented to being recruited as controls.

*Ethics*: The ethical committee of the hospital approved the study. Informed consent was obtained from both cases and controls, either from the subjects or their attendants prior to enrolment in the study.

### Determination of clinical and other characteristics:

Blood pressure (BP) was measured within 12 hours for cases and 1 hour for controls of hospital admission using standard protocol^26^^)^. The subjects were considered hypertensives if systolic (SBP) and diastolic blood pressures (DBP) were ≥140 mmHg and/or ≥90 mmHg respectively or if on antihypertensive treatment. Skinfolds (sum of triceps and sub-scapular skin-fold thicknesses) were measured^27^^)^ by Holtain’s skin-fold caliper. Diabetics were established cases of diabetes mellitus (DM) either on diet or drug treatment. Ischemic heart disease (IHD) was diagnosed when a history of angina pectoris or myocardial infarction was present or if there was electrocardiographic evidence of coronary heart disease (ST depression ≥1mV, abnormal T and Q waves). Two or more well trained physicians ascertained the presence of cardiac murmur or carotid bruit through auscultation. Family history of disease was considered positive if any member among first-degree blood relatives had the disease.

### Blood collection, storage, and determination of lipids and apoE genotypes:

The determination of blood lipids and apoE genotype was done at Tokyo Medical and Dental University, Japan. Nonfasting blood samples were collected in EDTA and sodium citrate containing vacuum tubes for both cases and controls at the hospital in Dhaka. Both EDTA, and citrated plasma (for measurement of fibrinogen) and cells were stored at -80°C until they were transferred on dry ice to Japan. DNA was isolated from peripheral blood leukocytes with Puregene (Gentra Systems, Inc).

Biochemical measurements were performed by autoanalizer (HITACHI 7170) within 3 months of blood collection. TC and HDL cholesterol were measured in accordance with the Lipid Standardization Program of the US centers for Disease Control and Prevention through the Osaka Medical Center for Cancer and Cardiovascular Diseases, Japan.

Determination of apoE genotype was performed by restriction enzyme digestion of an apoE polymerase chain reaction product^28^^)^.

### Statistical analyses:

All analyses were done by using the SAS statistical package (release 6.11, SAS Institute Inc). Comparisons of baseline characteristics between cases and controls were performed using Student’s t-test/ANCOVA for continuous variables, or simple/Mantel-Haenszel *χ*^2^ test for categorical variables with age (10-year age groups for categorical variables) and sex adjustments as applicable. The biochemical variables, along with BP levels across apoE genotypes, were adjusted for age, sex, and skinfolds, and compared by ANCOVA. The odds ratios (ORs) were adjusted for possible confounding effects of age, sex, skinfolds, smoking, hypertension, DM, and occupation by a conditional logistic regression model in which sex and age in 10-year increments were used for group matching, and the other variables were included as covariates. ORs for apoE *ε*2/*ε*2, *ε*2/*ε*3, *ε*3/*ε*4, and *ε*4/*ε*4 genotypes were calculated with *ε*3/*ε*3 as reference. To examine single-gene effects, ORs for *ε*2 carriers (*ε*2/*ε*2 and *ε*2/*ε*3) and *ε*4 carriers (*ε*3/*ε*4 and *ε*4/*ε*4) were also calculated with *ε*3/*ε*3 as reference. ApoE *ε*2/*ε*4 was excluded from the both above analyses as it was found only in 1 control. Since the relationship of apoE genotypes and stroke were different in the elderly and middle-aged individuals^22^^, ^^29^^)^, we examined the single gene effect in all ages, as well as by age group, having a cut off point at 60 years.

## RESULTS

### Participant Characteristics

As depicted in Table 1, the average age of the cases was lower than that of the controls. The ratio of males to females tended to be higher in cases than in controls. As expected, recognized risk factors, such as personal histories of hypertension, DM, and IHD in the infarction cases and only hypertension in the hemorrhage cases were more prevalent (P<0.01 for any comparisons) than in controls. Family histories of hypertension, IHD, and stroke, and higher skinfolds (P<0.01 for any comparisons) were also more frequent in cases than in controls.

**Table 1.** Comparisions of characteristics between cases with cerebral infarction or hemorrhage and controls.

	cerebral infarction n=147	cerebral hemorrhage* n=80	control n=190
Age, mean±sd	57.9±11.1 ‡	57.5±12.0	60.3±9.6
Male, %	79.9 ‡	79.0	67.7
Current smoker, %	32.0	31.5	26.9
Cardiac murmur, %	8.5	0	0
Carotid bruit, %	10.4	1.3	0
Atrial fibrillation, %	6.3	0	0
Hypertension, %	64.3 §	73.7 §	9.1
Diabetes mellitus, %	15.4 §	9.6	4.2
Ischemic heart disease, %	10.2 §	4.8	1.1
Family history of			
Hypertension, %	47.9§	62.5 §	15.5
Diabetes mellitus, %	14.0	13.5	9.9
Ischemic heart disease, %	7.6 §	5.7 §	0.5
Stroke, %	33.4 §	33.6 §	6.3
Skinfold thickness, mm †	28.6 §	30.2 §	25.6

Percentages (except male%, compared by chi square test) are adjusted for 10-year age groups and sex by indirect method and compared by Mantel-Haenzsel chi-square test; values are least square means±sem adjusted for age and sex.

* excludes subarachnoid hemorrhage.

† sum of triceps and subscapular skinfold thicknesses.

‡ P<0.05, and § P<0.01 as compared with the control group.

Table 2 shows biochemical characteristics and BP levels according to apoE genotype in the control group. TC in *ε*2/*ε*3 genotype (P=0.02) was lower than in *ε*3/*ε*3 genotype. However, other biochemical variables, and BP levels were not different among apoE genotypes in controls.

**Table 2.** Characteristics according to ApoE genotype in control group.

	Genotype*		

	*ε*2/*ε*3	*ε*3/*ε*3	*ε*3/*ε*4
Age, mean ± sd	61.2 ± 5.3	59.8(10.0)	62.0(8.7)
Male, %	66.7	68.5	75.9
TC, mg/dl	148.9 ‡	173.0	163.2
HDL-C, mg/dl	35.1	36.6	32.4 §
Fibrinogen, mg/dl	270.4	300.4	289.2
Fructosamne, µ mol/l	220.8	231.5	233.3
SBP, mm Hg †	130.1	129.0	136.1
DBP mm Hg †	78.3	77.2	79.0

Values are least square means adjusted for age, sex, and sum of triceps and subscapular skinfold thicknesses unless mentioned otherwise;

* Genotypes *ε*2/*ε*2, *ε*2/*ε*4, and *ε*4/*ε*4 were excluded from the analyses because of their too small sample size;

† subjects on antihypertensive medication have been excluded from the analysis.

§ P=0.02, and ‡ P=0.09 as compared with *ε*3/*ε*3.

### Allele and genotype frequencies

As shown in Table 3, there was no significant difference in the apoE genotypes or alleles frequencies between all strokes or its subtypes and controls. In cortical thrombosis cases, however, both *ε*3/*ε*4 genotype and *ε*4 allele occurred more frequently than in controls, although this was not statistically significant.

**Table 3.** ApoE genotype and allele distribution in stroke subtypes and control group.

	Genotypes							Alleles		
	
	n	*ε*2/*ε*2	*ε*2/*ε*3	*ε*2/*ε*4	*ε*3/*ε*3	*ε*3/*ε*4	*ε*4/*ε*4	*ε*2	*ε*3	*ε*4
Control	190	1.6	3.2	0.5	78.4	15.3	1.1	3.4	87.6	8.9
Stroke-all	227	1.3	2.2	0	78.9	16.7	0.9	2.4	88.3	9.3
Cerebral infarction-all	147	2.0	2.0	0	76.9	17.7	1.4	3.1	86.7	10.2
Cortical infarction	100	3.0	2.0	0	75.0	18.0	2.0	4.0	85.0	11.0
Thrombosis	54	1.9	1.9	0	75.9	18.5	1.9	2.8	86.1	11.1
Embolism	40	2.5	2.5	0	75.0	17.5	2.5	3.8	85.0	11.3
Penetrating infarction *	47	0	2.1	0	80.9	17.0	0	1.1	90.4	8.5
Cerebral hemorrhage †	80	0	2.5	0	82.5	15.0	0	1.3	91.3	7.5

We have failed to assign 6 cortical infarctions in either of the subtypes due to confusion ; Values are row percentages. In some cases, percentages may not sum to 100% due to rounding. No significant difference was observed in the genotype distribution or allele prevalence between the control and any of the case groups.

* region supplied by perforating arteries and includes internal capsule and basal ganglion.

† excludes subarachnoid hermorrhage.

### Apo E polymorphism and risk of stroke

Table 4 shows ORs (95%CI) in a conditional logistic regression model. The ORs of cortical thrombosis was 3.5 (95% CI 1.2 to 10.4) times greater for those with *ε*3/*ε*4 genotype than for those with *ε*3/*ε*3 genotype.

**Table 4.** Odds ratio* (95% confidence interval) of ApoE genotypes with stroke subtypes.

	n	Genotypes				

		*ε*2/*ε*2	*ε*2/*ε*3	*ε*3/*ε*3	*ε*3/*ε*4	*ε*4/*ε*4
All-stroke	227	1.3(0.1-13.5)	1.2(0.2-6.3)	1	1.6(0.8-3.3)	0.9(0.1-12.9)
Cerebral infarction-all	147	2.1(0.2-19.7)	0.7(0.1-5.1)	1	1.6(0.8-3.5)	1.3(0.1-16.6)
Cortical infarction	100	3.4(0.3-39.3)	0.4(0.1-3.2)	1	2.2(0.9-5.3)	2.2(0.2-31.5)
Thrombosis	54	4.5(0.3-80.8)	0.2(0.0-3.6)	1	3.5(1.2-10.4)§	1.0(0.0-88.6)
Embolism	40	1.5(0.0-49.8)	0.3(0.0-8.6)	1	2.0(0.6-6.6)	4.8(0.3-81.6)
Penetrating infarction †	47	…	1.8(0.1-22.3)	1	1.3(0.4-4.1)	…
Cerebral hemorrhage ‡	80	…	2.1(0.2-19.2)	1	1.9(0.7-5.7)	…

… Indicates no cases.

* Analysed by conditional logistic regression model with group matching for sex and age in 10-year band and adjusted for skinfolds, smoking, hypertension, diabetes mellitus and occupation. ApoE *ε*2/*ε*4 was excluded from the analyses.

† ‡ same as table3.

§ P=0.02.

Table 5 shows the association of *ε*2 and *ε*4 carriers with stroke in different age groups. *ε*4 carriers had approximately 3 fold increased risk of cortical thrombosis (OR 3.1 95% CI 1.1 to 9.0) in all age group, and *ε*2 carriers risked hemorrhagic stroke (OR 19.2 95% CI 1.3 to 295.2) in age group >60 years, compared with *ε*3/*ε*3 carriers.

**Table 5.** Odds ratio* (95% CI) of ApoE alleles in stroke subtypes categorised by age group.

	n	All age group		n	≤60 years		n	>60 years	
		
		*ε*2 carrier	*ε*4 carrier		*ε*2 carrier	*ε*4 carrier		*ε*2 carrier	*ε*4 carrier	
All-stroke	227	1.2(0.3-4.9)	1.6(0.8-3.1)	131	0.3(0.0-2.4)	1.4(0.5-4.0)	95	3.8(0.7-21.7)	2.0(0.7-5.7)	
Cerebral infarction	147	1.1(0.3-4.9)	1.6(0.8-3.3)	84	0.6(0.1-5.1)	1.2(0.4-3.9)	63	3.2(0.5-21.8)	2.2(0.7-6.6)	
Cortical infarction	100	1.0(0.2-4.9)	2.2(0.9-5.0)	54	0.7(0.1-7.8)	1.5(0.3-6.7)	46	2.8(0.3-25.7)	2.9(0.9-9.7)	
Thrombosis	54	0.8(0.1-5.9)	3.1(1.1-9.0)§	27	0.6(0.0-11.1)	3.6(0.4-30.1)	27	2.5(0.2-36.4)	3.8(0.9-15.9)	
Embolism	40	0.7(0.1-6.7)	2.2(0.7-6.7)	23	1.3(0.1-19.8)	1.2(0.2-7.1)	17	…	1.9(0.3-12.5)	
Penetrating infarction †	46	0.9(0.1-10.1)	1.2(0.4-3.8)	30	…	1.0(0.2-5.5)	17	5.7(0.2-150.2)	1.4(0.2-10.8)	
Cerebral hemorrhage ‡	80	1.2(0.2-8.7)	1.9(0.6-5.4)	47	…	2.5(0.5-12.3)	33	19.2(1.3-295.2)∥	1.8(0.3-10.5)	

… Indicates no cases.

* Analysed by conditional logistic regression model with group matching for sex and age in 10-year band and adjusted for skinfolds, smoking, hypertension, diabetes mellitus and occupation. ApoE *ε*2/*ε*4 was excluded from the analyses. Genotypes containing *ε*2 or *ε*4 allele are *ε*2 or *ε*4 carrier respectively. ApoE *ε*2/*ε*4 was excluded from the analyses.

* † ‡ Same as table 3.

§ ∥ P =0.03.

## DISCUSSION

We studied the association of apoE genetic polymorphism with stroke subtype in a Bangladeshi hospital-based population, which is the first of its type in South Asian countries. In this study first-ever-stroke patients were employed and classified with CT documentation. The wide age range of subjects, who were recruited in the acute phase of stroke, allowed us to include fatal cases in our study and to perform age specific analysis.

We found that, independent of other traditional risk factors, carrying a *ε*4 allele was associated with an increased risk for cortical thrombosis. No association of *ε*4 allele was found out with infarction in penetrating artery region. These findings suggested that the association of apoE *ε*4 allele with ischemic stroke was confined to thrombosis in the brain area supplied by the large cortical artery. Our results were compatible with 2 case-control studies^19^^, ^^30^^)^, where in subtype analysis the investigators detected *ε*4 allele to be associated with large vessel disease, but are inconsistent with others^14^^-^^18^^)^. These inconsistencies are likely to be due to methodological differences, i.e., in classifying stroke, the inclusion of only elderly subjects^14^^-^^16^^)^, and small sample sizes^14^^, ^^18^^)^. Two other case-control studies^13^^, ^^20^^)^, which demonstrated positive association of *ε*3/*ε*4 with ischemic stroke used non-fatal cases in risk analyses. Among three cohort studies^22^^-^^24^^)^, the non- detection of any association with apoE genetic polymorphism probably resulted from not taking into consideration heterogeneous pathogenesis in differing stroke subtype. Moreover, two of the cohort studies^22^^, ^^24^^)^ used elderly subjects (average age >78 years) and the other^23^^)^ had a small sample size.

The age distribution of the population of cases and controls is an important confounding factor because risk factor profiles of ischemic stroke differ between young and elderly stroke patients^31^^)^ and apoE allele frequencies differ among age groups in the normal population^29^^)^. Age-dependent association of *ε*4 (<70 years)^13^^, ^^19^^-^^21^^)^ and *ε*2 (>70 years)^15^^, ^^30^^)^ carriers with stroke as reported by previous studies suggested that the two alleles might act differently in stroke etiology and possibly with different time frames over the life span. Studies on the relationship between apoE polymorphism and survival showed a decreasing survival across *ε*4, *ε*3, and *ε*2 carriers which was ascribed to the effect of apoE isoforms on atherosclerosis and perhaps dementia.^29^ ApoE *ε*4 and *ε*2 allelic frequencies tended to decline and to increase with age, respectively^29^^)^. The mean ages of ischemic and hemorrhagic stroke in the present study were 57.9 and 57.5 years respectively. Our results supported positive associations between *ε*4 carriers and cortical thrombosis in younger individuals. In our population, however, *ε*2 carriers had an increased risk of cerebral hemorrhage when the analysis was restricted for age group >60 years which supported a previous Japanese study^30^^)^ where *ε*2 had increased risk for Intracerebral hemorrhage.

Analysis according to stroke subtype is important to gain insight into the mechanisms underlying different stroke types. Ischemic stroke subtypes, as in the cortical and penetrating artery regions have different pathogenic mechanisms involved in cerebral ischemia. Thrombotic stroke resulting from large vessel disease usually is the consequence of underlying atherosclerotic process which is chiefly related to lipid levels^32^^)^, whereas infarction in penetrating artery region presumably have other common pathological features: arteriolosclerosis or angionecrosis^33^^)^, which is strongly associated with hypertension but not with hypercholesterolemia or obesity^1^^)^. We do not refer to the difference in lipid levels between cases and controls in the present study, because the potential cause-effect reversal of biochemical variables limits the accuracy of case-control studies^34^^)^. Thereby, we can not firmly say whether any association of the apoE gene with stroke is independent of lipid levels. In a separate analysis, however, with additional adjustment by TC and HDL cholesterol (data not shown) in the multivariate models, weaker but still positive associations persisted for the both *ε*3/*ε*4 genotype and *ε*4 carrier with cortical thrombosis. But, the positive association with cerebral hemorrhage in the elder group disappeared. This result suggested that the apoE gene might be associated with cerebral hemorrhage not directly, but rather through its influence over lipids or the underlying process that leads to cerebral hemorrhage in the elderly. On the other hand, the association of apo-E gene with cortical thrombosis was found to be independent of lipids. However this result should be interpreted with great caution because changes in lipid levels after stroke have been observed in different studies^34^^-^^37^^)^.

As expected, in the present control population (Table 2), *ε*2/*ε*3 subjects had lower level of TC and *ε*3/*ε*4 subjects had lower level of HDL cholesterol than *ε*3/*ε*3 subjects. However, the preponderance of *ε*4 alleles in both coronary heart disease and ischemic stroke remains to be resolved. In the both Multiple Risk Factor Intervention Trial^38^^)^ and Framingham Offspring study^11^^)^, the persistence of significant association of *ε*4 allele with coronary heart disease adjusted for LDL cholesterol further adds to this complexity, suggesting that *ε*4 allele effect may be independent of lipid levels. This was also demonstrated in carotid atherosclerosis^13^^)^. The isoform-specific antioxidant activity^39^^)^, platelet cNOS activity and other mechanisms^40^^)^ may account for the atherogenic nature of *ε*4 allele. Moreover, apoE *ε*4 was reported to be more strongly associated with vascular deposition of β-amyloid peptide than the other apoE genotypes^41^^)^, the carrier of which have the highest relative risk for cortical microinfarcts and hemorrhage but multiple lesion types are often present (e.g., larger cortical infarcts and lacunes)^42^^)^. However, we do not possess data whether the present stroke patients were suffering from amyloid angiopathy or not. Conversely, *ε*2 carriers may be prone to intracerebral hemorrhage by producing endothelial weakening of the arteries as a result of lower cholesterol levels^43^^, ^^44^^)^. In this study, the association between *ε*2 carriers with cortical hemorrhage was demonstrated only among the elderly but not in the younger. However, the differences of lifestyle in the younger due to generation gap, as reflected by higher skinfolds and TC levels (data not shown) than the elder, might had masked the possible association.

This study in a Bangladeshi hospital conforms to the classical risk factors of stroke (Table 1) which is already established in the Western and Japanese population. In the western populations the etiology of embolic infarction is almost solely explained by concomitant IHD, whereas in Japan it is mainly due to long-term hypertension or congestive heart failure or insufficiency^1^^)^. The 29% prevalence of IHD in the cerebral embolism cases in our study makes it etiologically much closer to the Japanese.

The frequency of *ε*4 allele in our controls was 0.89, which was lower than in Caucasians but closer to the Japanese levels^37^^)^. We found both apoE *ε*3/*ε*4 genotype and *ε*4 allele frequencies to be more common (non-significant) in cases with cortical thrombosis than in controls. However, multivariate logistic regression analyses adjusted for potential confounders revealed a positive association of both apoE *ε*3/*ε*4 genotype and *ε*4 carriers with cortical-thrombosis.

The major limitations of this study were firstly, the selection of idiopathic cataract patients as control group. The possibility of an independent interaction of apoE genetic polymorphism with this condition was not excluded. Secondly, we did not routinely perform specific investigations for conclusive detection of cardiac sources of embolism and atherosclerotic plaque in large vessels. Finally, the results of analyses in the younger and elder groups might be biased by relatively small sample size.

In conclusion, our data suggested that carriers of apoE *ε*4 allele were more at risk of developing thrombotic stroke in the cortical artery territory in a Bangladeshi hospital-based study. Meanwhile, a positive age-dependent effect between *ε*2 and risk of hemorrhagic stroke was also observed in the elderly. The consistency of our results according to type-specific analysis further needs to be assessed in cohort or nested case-control studies in different racial populations.
